# Implementation and acceleration of optimal control for systems biology

**DOI:** 10.1098/rsif.2021.0241

**Published:** 2021-08-25

**Authors:** Jesse A. Sharp, Kevin Burrage, Matthew J. Simpson

**Affiliations:** ^1^ School of Mathematical Sciences, Queensland University of Technology, Brisbane, Australia; ^2^ ARC Centre of Excellence for Mathematical and Statistical Frontiers, Queensland University of Technology, Brisbane, Australia; ^3^ Department of Computer Science, University of Oxford, Oxford OX2 6GG, UK

**Keywords:** convergence acceleration, forward–backward sweep method, optimal control, Wegstein, Aitken–Steffensen, Anderson

## Abstract

Optimal control theory provides insight into complex resource allocation decisions. The forward–backward sweep method (FBSM) is an iterative technique commonly implemented to solve two-point boundary value problems arising from the application of Pontryagin’s maximum principle (PMP) in optimal control. The FBSM is popular in systems biology as it scales well with system size and is straightforward to implement. In this review, we discuss the PMP approach to optimal control and the implementation of the FBSM. By conceptualizing the FBSM as a fixed point iteration process, we leverage and adapt existing acceleration techniques to improve its rate of convergence. We show that convergence improvement is attainable without prohibitively costly tuning of the acceleration techniques. Furthermore, we demonstrate that these methods can induce convergence where the underlying FBSM fails to converge. All code used in this work to implement the FBSM and acceleration techniques is available on GitHub at https://github.com/Jesse-Sharp/Sharp2021.

## 1. Introduction

Across the life sciences, we encounter systems over which we wish to exert control. Whether we consider outbreak control in epidemiology [1,2], chemotherapy in oncology [3–5], muscle contraction and gait regulation in biomechanics [6–8], engineering cellular processes in synthetic biology [9,10], cell population growth in tissue engineering [11,12], or biodiversity and invasive species management in ecology [13–15], we face decisions around how a particular intervention should be applied to best achieve desired outcomes. Using mathematical models of such systems, optimal control theory provides insight into these resource allocation decisions.

**Figure 1.**
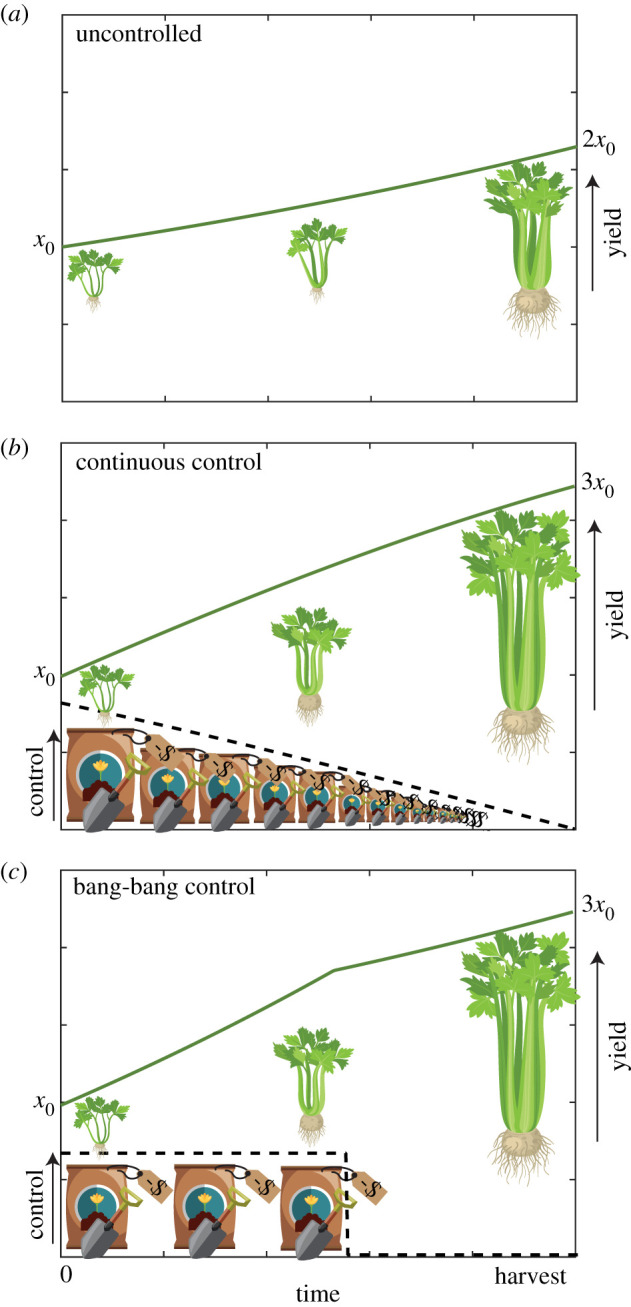
A pictorial example of optimal control for a growing crop. Suppose that initially, the crop yield is *x*_0_. We want to grow this crop to increase the yield, represented by the green line, come harvest time. Actions taken to increase the growth rate of the crop, such as applying fertilizer, are the controls, represented in black dash. Scenarios are presented for (*a*) no control, (*b*) continuous control and (*c*) bang-bang control. Optimal control theory helps us determine how best to apply these controls. Illustrations adapted from ilyakalinin/iStock/Getty Images, johavel/iStock/Getty Images.

Optimal control is a science of trade-offs; between competing objectives, or in weighing up the benefits of control measures against their costs. We illustrate some key concepts of optimal control in figure 1. Suppose that without intervention, a crop yield will double, from *x*_0_ to 2*x*_0_, between now and harvest time. We might consider applying a control, such as fertilizer, to increase the growth rate of the crop; thereby increasing the yield at harvest to 3*x*_0_. Of course, applying fertilizer comes at a cost, and this must be considered against the increase in crop yield. As such, it is not immediately apparent how much fertilizer should be applied, and over what time period. This depends entirely on our characterization of optimality: the *pay-off* . Depending on the pay-off, the optimal control may be continuous; whereby the strength can be readily and continuously adjusted throughout time, or bang-bang (discontinuous); whereby the control is applied at either a lower or upper bound with finitely many discrete switches between the two. The pay-off determines the objective(s) of control; which in our stylized example may be to maximize profits after cost of fertilizing is considered, or achieve a specific yield, for example 3*x*_0_, using the minimum amount of fertilizer.

Much of modern day optimal control theory stems from the seminal works of Pontryagin, through the Pontryagin maximum principle (PMP) [16], and Bellman, through the advent of dynamic programming and the Hamilton–Jacobi–Bellman equation [17], in the 1950s and 1960s. These foundations of optimal control are built upon centuries of development in the calculus of variations [18]. For brief but broad expositions of the theoretical roots of optimal control and developments following these seminal works, we direct the reader to articles such as [19,20].

Often we are unable to solve optimal control problems analytically, so we pursue computational approaches. Broadly, the numerical methods for optimal control can be classed as either indirect or direct methods; for indirect methods optimality conditions are derived in the calculus of variations fashion via the PMP, leading to a two-point boundary value problem (TPBVP), while for direct methods the control problem is discretized and reformulated as a nonlinear programming problem [21]. For an early history of numerical methods in optimal control, including gradient and conjugate gradient methods, Newton–Raphson methods, quasi-linearization, feasible direction algorithms and feedback solutions we suggest [22]. Surveys [20,21] give an excellent overview of more recent developments in relevant numerical methods, including the forward–backward sweep method (FBSM), multiple-shooting methods, control parameterization, collocation and pseudospectral methods and complete discretization into finite-dimensional nonlinear programming problems.

Optimal control methodology and numerical solution techniques are continually being developed and improved. The growing popularity of artificial intelligence, machine learning and related disciplines has precipitated significant advances in computational techniques for handling large-scale systems with many variables, and related infinite-dimensional optimization problems. Nonlinear approximators, including neural networks, can be used to reduce infinite-dimensional optimization problems to finite-dimensional nonlinear programming problems. This approach is presented in [23], alongside other techniques that arise through unifying aspects of decision science, dynamic optimization, statistical and deterministic machine learning, nonlinear approximation theory and other fields. One example of control paired with machine learning arises in autonomous vehicles, where machine learning techniques can accelerate obtaining approximately optimal controls where computational power on-board is limited and controls satisfying strict safety constraints must be obtained rapidly [24]. Reinforcement learning, a technique from artificial intelligence resembling a model-free analogue of dynamic programming, has shown promising simulation results for the control of multi-species microbial communities in bioreactors [25].

Formulation and approximate solutions of fractional optimal control problems (FOCP)—optimal control of systems involving fractional derivatives—has also garnered wide interest recently within the control, numerical methods and applied mathematics communities; resulting in the development of new numerical approaches such as the non-standard two-step Lagrange interpolation method [26,27]; and amalgamations of new and existing techniques, such as pairing predictor-corrector methods for solving fractional differential equations with the FBSM for optimal control [28,29]. Applications involving FOCPs arise in areas of systems biology including epidemiology, where the incorporation of memory effects through fractional time derivatives may better describe disease transmission, by modelling the capacity for the population to learn from past outbreaks [26,30]; and in cancer therapy for determining optimal chemotherapeutic and immunotherapeutic treatment regimens [31,32].

The field of optimal control has historically focused on determining optimal interventions to apply to systems to meet specified objectives. More recently, however, optimal control techniques have been applied in a systems biology context to further our understanding of the underlying mechanisms or processes involved in a given system; for example via inverse optimal control, whereby exhibited behaviour observed in a system is used to elicit the underlying optimality principles that may guide the system [33]. Optimality principles have been employed to investigate mechanisms in metabolism; for example, in [34], where optimal control techniques provide rationalization for experimentally and numerically observed sequential activation of metabolic pathways; in [35] where optimal control techniques predict enzyme activation times and metabolite concentrations; and in other work reviewed in [36], where further insights are gained regarding metabolic pathway activation and regulation. Optimal control has also provided insight into the emergence of persister cells in the presence of environmental volatility [37].

The FBSM is an iterative method for solving the TPBVPs that arise from the indirect PMP approach to optimal control. In systems biology, the FBSM for optimal control is very popular, owing particularly to its straightforward scalability to large systems, and to its moderate computational cost and mathematical complexity [38]. In this work, we review the implementation of the FBSM to solve optimal control problems, and investigate means of accelerating the convergence. To contextualize our discussion of the FBSM, we first consider the more familiar technique of successive over-relaxation (SOR). SOR is a generalization of the Gauss–Seidel method, and is widely applied in numerical linear algebra to accelerate convergence when solving linear systems iteratively [39]. Essentially, the process of SOR involves specifying an acceleration or relaxation parameter, *β* ∈ (0, 2); a weighting factor that serves to reduce the spectral radius of the iterative matrix operator [40]. The error and rate of convergence of SOR is sensitive to this (problem dependent) choice of *β*, prompting investigation into theoretical convergence results and methods of determining *β* [40–42]. Despite challenges in identifying the optimal *β*, the SOR has historically been widely applied and studied in the literature due to the ease with which it can be implemented, and the rapid convergence it can deliver; even without identifying the optimal *β* [43,44].

This narrative closely parallels that of the FBSM in optimal control, where a weighting factor *ω* can be applied when updating the control between iterations to aid convergence. The optimal choice of *ω* is problem dependent, and significantly impacts the rate of convergence, or whether the FBSM converges at all. Nonetheless, the FBSM is frequently used in applied optimal control work as it is relatively straight-forward to implement, and can still converge in absence of the optimal *ω*. Theoretical convergence results of the FBSM are available in the literature [45,46], although the focus is on the FBSM without weighted updating, with no consideration for choosing *ω*. Using regularization techniques, the FBSM is modified in [47] to improve convergence properties for large systems in a continuous setting, with a view to training deep neural networks in machine learning. These convergence results have recently been extended to the numerically discretized setting through symplectic Runge–Kutta discretization; taking advantage of the variational structure of optimal control problems [48]. The authors also demonstrate that the rate of convergence of the regularized FBSM with symplectic discretization can be improved with Anderson acceleration, an iterative acceleration technique. Although promising, this regularization introduces a regularization parameter, *ρ*. Similar to *ω*, the choice of *ρ* impacts convergence, and its choice is problem dependent. Understanding and implementing the regularization and symplectic techniques is not trivial, and introduces conceptual complexity beyond what is necessary for many applied optimal control problems. As such, the standard FBSM remains an attractive choice for practitioners.

**Figure 2.**
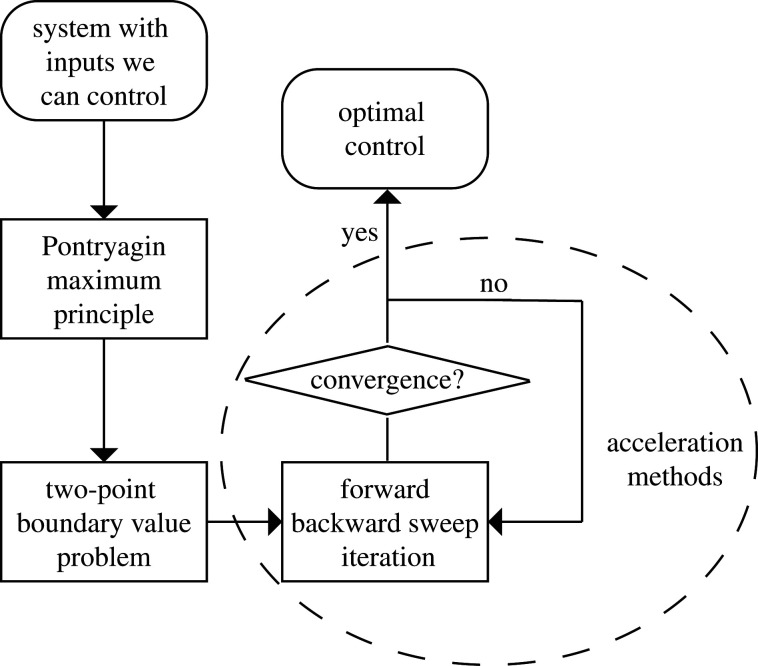
The process of optimal control via the Pontryagin maximum principle approach, with the incorporation of acceleration methods.

To this end, we aim to review acceleration techniques that can be paired with the standard FBSM. We implement such techniques alongside the FBSM with the goals of: (1) increasing the rate and frequency of convergence and (2) reducing the importance of, and challenges associated with, selecting *ω*. A graphical overview of the optimal control process we employ in this work, including the incorporation of acceleration methods, is presented in figure 2. We note that all code used to implement the algorithms presented in this review, the FBSM and the Wegstein, Aitken–Steffensen and Anderson acceleration methods, is available on GitHub (https://github.com/Jesse-Sharp/Sharp2021).

Throughout this work, we consider optimal control in the systems biology context. However, we note that optimal control is relevant to a wide variety of fields including chemical engineering [49], aeronautics and astronautics [21], management science and economics [50]. The FBSM, and by extension, the acceleration techniques we consider in this work, can be readily applied in any of these areas.

In §2, we review the PMP approach to optimal control, and the implementation of the FBSM. We provide a single-variable linear model, and a multi-variable nonlinear model in §3; and pose and solve example continuous, bang-bang (discontinuous), and fixed endpoint control problems. We review potential iterative acceleration methods in §4, and present the results of selected techniques in §5. We discuss the performance of these techniques in §6, and identify opportunities for application and further investigation.

## 2. Forward–backward sweep method

In an optimal control problem with one state variable, *x*(*t*), one control, *u*(*t*), over a fixed time interval, *t* ∈ [*t*_0_, *t*_*N*_], such as the crop growth example presented in figure 1, we seek the optimal control u*(*t*) that minimizes or maximizes a specified pay-off function, *J*, subject to the dynamics of the state. In this section, we briefly review the PMP approach to such an optimal control problem, and the standard implementation of the FBSM for solving the resulting two-point boundary value problem. The FBSM is readily extended to problems with multiple state variables, multiple controls, state constraints and free end-times [5,38,46,51]; however for this overview, we restrict ourselves to the single variable, single control, fixed end-time case for clarity.

The pay-off typically comprises a cost function L(t,x(t),u(t)) integrated over the time interval, and/or a function, *ϕ*, of the state at final time: *ϕ*(*x*(*t*_*N*_)). As such, we seek to minimize or maximize *J*, subject to

2.1 $$J=\phi (x(t_{N}))+\int_{t_{0}}^{t_{N}}L(t,x(t),u(t))\;dt$$

and

2.2 $$\frac{dx}{dt}=f\;(x(t),u(t),t),\;x(t_{0})=x_{0}.$$

Applying the PMP, we construct the Hamiltonian H(t,x(t), u(t),λ(t))=L(t,x(t),u(t))+λ(t)f (x(t),u(t),t), where *λ*(*t*) is the co-state variable linking our state to our pay-off. The necessary conditions for optimal control are obtained from the Hamiltonian:

(1) The optimal control, u*(*t*), is obtained by minimizing the Hamiltonian
  2.3 $$\frac{\partial H}{\partial u}=0.$$
(2) The co-state is found by setting
  $$\frac{d\lambda }{dt}=-\frac{\partial H}{\partial x},$$
(3) satisfying the transversality condition
  2.4 $${\lambda (t_{N})=\lambda_{N}=\frac{\partial \phi }{\partial x}|}_{t=t_{N}}.$$

Following these steps yields a TPBVP to solve for *x*(*t*), *λ*(*t*), subject to *x*(*t*_0_) = *x*_0_, and *λ*(*t*_*N*_) = *λ*_*N*_. To solve this numerically, we discretize *t* into *N* + 1 time points separated by a step-size d*t* = (*t*_*N*_ − *t*_0_)/*N*; **t** = [*t*_0_, *t*_0_ + d*t*, …, *t*_0_ + *N*d*t*] = [*t*_0_, *t*_1_, …, *t*_*N*_]. Here, we consider a uniform discretization in time; although this is not strictly necessary, as discussed in §3. Using superscripts to denote the iteration number, provide an initial guess of the control at each *t*; $u^{\left(0\right)}=[u_{0}^{\left(0\right)},u_{1}^{\left(0\right)},\ldots ,u_{N}^{\left(0\right)}]$. From **u**^(0)^, solve equation (2.2) numerically from *t*_0_ to *t*_*N*_ to obtain $x^{\left(0\right)}=[x_{0}^{\left(0\right)},x_{1}^{\left(0\right)},\ldots ,x_{N}^{\left(0\right)}]$. Now, using **x**^(0)^, solve for $\lambda^{\left(0\right)}=[\lambda_{0}^{\left(0\right)},\lambda_{1}^{\left(0\right)},\ldots ,\lambda_{N}^{\left(0\right)}]$ backwards in time from *t*_*N*_ to *t*_0_, starting from *λ*_*N*_. With the optimality condition from equation (2.3), generate a temporary update for the control, ${\hat{u}}^{\left(1\right)}$. The next iteration begins with an updated guess for the control, **u**^(1)^. These steps are repeated until a convergence condition is satisfied. The algorithm for the FBSM is summarized in §1 of the electronic supplementary material.

In some instances, directly updating the control, such that

2.5 $$u^{\left(k\right)}={\hat{u}}^{\left(k\right)},\;k=1,2,\ldots$$

is sufficient; however more commonly a weighted update is performed [5,38], such that in the (*k* + 1)th iteration,

2.6 $$\begin{array}{rl} & u^{\left(k+1\right)}=\omega u^{\left(k\right)}+(1-\omega ){\hat{u}}^{\left(k+1\right)},\\ & \;k=1,2,\ldots ,\omega \in [0,1). \end{array}$$

This weighted updating is also referred to as applying a relaxation factor, similar to SOR as discussed in §1. An appropriate choice of *ω* in equation (2.6) can accelerate convergence relative to equation (2.5), or in some cases induce convergence where equation (2.5) leads to divergence. The weighting parameter, *ω*, can be held constant between iterations, although faster convergence may by achieved by updating *ω*. For example, by reducing *ω* as the system approaches convergence, a greater portion of the updated control is maintained relative to the control from the previous iteration [38], possibly accelerating convergence. A challenge commonly faced in implementing this control updating scheme is that the best choice for *ω* is problem dependent, and often is determined heuristically in practice. We address the extent to which the proposed acceleration algorithms address this issue in §4.

To facilitate the following discussion regarding acceleration, we note that the FBSM can be thought of as a generalized fixed point iteration [46], where each iteration comprises a forward and backward sweep and a control update. As such, for a control problem with one control, discretized into *N* + 1 time points, each iteration of the FBSM can be thought of as the application of a nonlinear operator, F, of dimension *N* + 1, such that $u^{\left(k+1\right)}=F(u^{\left(k\right)})$, or:

$$\left[\begin{array}{c} u_{0}^{\left(k+1\right)}\\ u_{1}^{\left(k+1\right)}\\ \vdots \\ u_{N}^{\left(k+1\right)} \end{array}\right]=\left[\begin{array}{c} \;f_{0}(u_{0}^{\left(k\right)},u_{1}^{\left(k\right)},\;\ldots ,u_{N}^{\left(k\right)})\\ f_{1}(u_{0}^{\left(k\right)},u_{1}^{\left(k\right)},\;\ldots ,u_{N}^{\left(k\right)})\\ \vdots \\ f_{N}(u_{0}^{\left(k\right)},u_{1}^{\left(k\right)},\;\ldots ,u_{N}^{\left(k\right)}) \end{array}\right],$$

where $F={\left(f_{0},f_{1},\;\ldots ,\;f_{N}\right)}^{T}$. However, in general, we are not able to write down an explicit expression for F. Viewing the FBSM as a fixed point iteration process informs the choice of acceleration methods discussed in §4.

Importantly, we use the term *function evaluation* in this work to refer to the process of solving the system of ODEs for the state forward in time and the system of ODEs for the co-state backwards in time, once. This aligns with a single iteration of the standard FBSM. The function evaluation nomenclature becomes convenient when discussing the FBSM in the context of acceleration algorithms that typically focus on reducing the number of times expensive functions are evaluated. Producing numerical solutions to the ODE systems is by far the most computationally expensive component of the FBSM. This computational expense increases with the size and complexity of the systems; reducing the number of times these systems must be solved becomes more advantageous as the size and complexity of the systems increases. The function evaluation description also facilitates comparison between acceleration methods that require solving the ODE systems a different number of times per iteration. Throughout this work, we use N to denote the total number of function evaluations a given method takes to achieve convergence.

### 2.1. Adapted forward–backward sweep method

The FBSM can be extended to handle problems where we aim not only to minimize or maximize a given quantity over time but also ensure that a specific state is reached at final time. This aligns with the crop growth example from figure 1 if the objective is to achieve a specific yield of 3*x*_0_ at harvest, rather than to maximize yield. In this case, we may have an integral term in the pay-off as described in equation (2.1); however, the function of the final state, *ϕ*(*x*(*t*_*N*_)), is redundant in a control problem with a prescribed final state. Equation (2.2) is also modified to incorporate the additional constraint

2.7 $$\begin{array}{rl} & J=\int_{t_{0}}^{t_{N}}L(t,x(t),u(t))\;dt,\;\mathrm{subject}\;\mathrm{to}\;\\ & \frac{dx}{dt}=f\;(x(t),u(t),t),\;\;x(t_{0})=x_{0},\;x(t_{N})=x_{N}. \end{array}$$

Here, *x*_*N*_ is the specified state that must be reached at final time. Since we have introduced an additional boundary value to the system, we no longer obtain the transversality condition from equation (2.4). Instead, we seek the final time condition on the co-state, *λ*_*N*_, and associated optimal control that satisfies equation (2.7). We proceed by considering an adapted FBSM that takes as an input a guess for this final time condition, ${\hat{\lambda }}_{N}$, and solves the corresponding control problem. If we denote this application of the FBSM as the function $V({\hat{\lambda }}_{N})$, and the corresponding final value of the state, ${\hat{x}}_{N}$, then the adapted FBSM is an iterative process that solves for the root of $V({\hat{\lambda }}_{N})$; the value of $\hat{\lambda_{N}}$ for which $x_{N}-{\hat{x}}_{N}=0$. This outer iterative process can be solved using standard techniques such as the bisection method or secant method; the former converging more reliably provided that the initial guesses for ${\hat{\lambda }}_{N}$ form an interval that brackets the root, the latter converging in fewer iterations [38]. Each of these outer iterations necessitates solving a boundary value problem to convergence, often involving numerous iterations of the FBSM. In this work, we apply the secant algorithm as presented in [38] without modification, for the adapted FBSM. The acceleration techniques described in §4 are applied only to the inner FBSM processes, reducing N for each internal FBSM problem, leaving the outer secant iterations unchanged. Using $N^{\left(k\right)}$ to denote the number of function evaluations in the *k*th internal FBSM problem, we can express the cumulative function evaluations required for convergence of the adapted FBSM as Σ, such that $\Sigma =N^{\left(1\right)}+N^{\left(2\right)}+\cdots$.

The adapted FBSM can also be used to solve control problems with isoperimetric constraints; integral constraints of the form

$$\int_{t_{0}}^{t_{N}}h(t,x(t),u(t))\;dt=K,$$

where *K* is a prescribed constant. For example, if *h*(*t*, *x*(*t*), *u*(*t*)) = *u*(*t*), then *K* represents a specific and known amount of control that must be applied. The approach to solve problems with isoperimetric constraints, as outlined in [38], is to introduce an additional state variable, *z*, with

$$\frac{dz}{dt}=h(t,x(t),u(t)),\;z(t_{0})=0,\;z(t_{N})=K.$$

This transforms the problem with an isoperimetric constraint into a problem with a fixed endpoint, that can be solved using the adapted FBSM as described.

## 3. Control problems

To investigate the robustness and effectiveness of the iterative acceleration techniques that we will discuss in §4, we consider two distinct systems, and for each system we study three example control problems. The first system is a single species linear differential equation subject to a control. We later demonstrate that under certain conditions we are able to obtain exact solutions for control problems applied to this model. The second system is a three species model for acute myeloid leukaemia (AML) governed by a coupled nonlinear system of differential equations, subject to a control. We construct the linear model to examine the behaviour of the acceleration techniques as applied to a simple idealized set of control problems. We include the AML model, variations upon which have been considered in the literature [5,51,52], to examine how the acceleration techniques perform when applied to problems more reflective of those considered in applied optimal control. For each model, we consider three distinct control problems: continuous control, bang-bang control and continuous control with fixed endpoint.

For all control results presented in this work, convergence is deemed to be achieved when the error, ɛ, measured as the Euclidean norm of the difference between subsequent controls, falls below a tolerance of 1 × 10^−10^. Numerical solutions to ODEs are obtained using a fourth-order Runge–Kutta method [53] with constant time-stepping. A uniform time discretization is sufficient for all control problems considered in this work. However, the FBSM and acceleration methods readily generalize to a non-uniform discretization. If the desired discretization for the state equations differs from that of the co-state equations, it is necessary to perform interpolation within each iteration of the FBSM to obtain values at corresponding time points. This can be computationally expensive and introduce an additional source of error. Where the desired discretizations for the state and co-state differ, numerical schemes with internal interpolation such as continuous Runge–Kutta methods may be appropriate [54,55].

### 3.1. Single-variable linear model

The linear model is a single species model for the growth of *x*(*t*), subject to control *u*(*t*) that increases the growth rate. This model could represent our stylized crop growth example presented in §1. We suppress the explicit time dependence of the state and co-state variables and the control in the following equations for notational convenience. For numerical results, we solve the linear problems on the domain 0 ≤ *t* ≤ 1, with time-step d*t* = 3.91 × 10^−3^, giving *N* = 257 time points.

3.1 $$\frac{dx(t)}{dt}=\gamma x(t)+u(t),\;x(0)=x_{0},\;\gamma >0,\;0\leq t\leq 1.$$

In the absence of control, *u*(*t*) ≡ 0, this model admits the solution *x*(*t*) = *x*_0_ e^*γt*^, describing exponential growth.

#### 3.1.1. Continuous control

We seek to maximize a quadratic cost function *J*, subject to

3.2 $$J=\int_{0}^{1}(ax^{2}-bu^{2})\;dt,\;a>0,\;b>0.$$

Following the standard PMP approach for solving optimal control problems, we form the Hamiltonian and derive the co-state equation, transversality condition and optimality condition. The Hamiltonian is given by

3.3 $$H=ax^{2}-bu^{2}+\lambda (\gamma x+u).$$

The co-state equation is

3.4 $$\frac{d\lambda }{dt}=-\frac{\partial H}{\partial x}=-2ax-\lambda \gamma ,$$

with transversality condition *λ*(1) = 0. In this case, the optimality condition is

$$\frac{\partial H}{\partial u}=\lambda -2bu=0,$$

such that the optimal control is given by

3.5 $$u^{*}(t)=\frac{\lambda (t)}{2b}.$$

**Figure 3.**
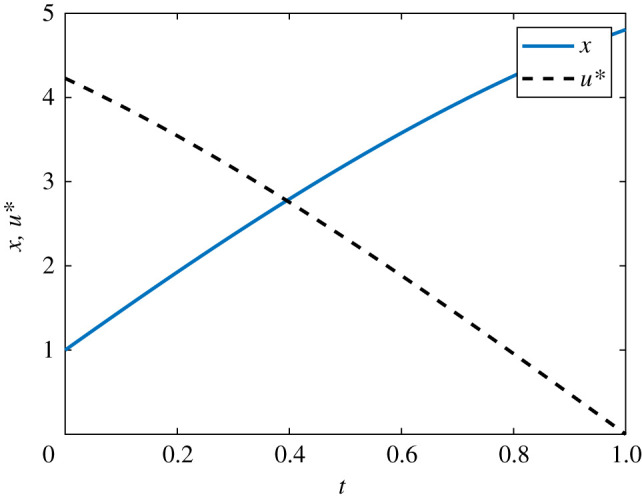
Solution to the linear continuous control problem. The optimal control, u*(*t*), is shown in black dash and the corresponding state, *x*(*t*), in blue. This solution is produced with model parameter *γ* = 0.5, time-step d*t* = 3.91 × 10^−3^, over the interval 0 ≤ *t* ≤ 1. The contributions of the state and the control to the pay-off are equally weighted, with *a* = *b* = 1.

For model parameter *γ* = 0.5 and pay-off weightings *a* = *b* = 1, with initial condition *x*_0_ = 1, we are able to solve the control problem analytically using standard techniques for linear systems with complex eigenvalues [56]. The process is laborious so we present the approach and analytical solution in §2 of the electronic supplementary material. In the electronic supplementary material, we also plot the analytical results against the numerical results to demonstrate the excellent agreement. The numerical solution to the linear continuous control problem is presented in figure 3. Convergence via the FBSM requires N=57 iterations.

#### 3.1.2. Bang-bang control

For the bang-bang control, we consider the same state equation as in equation (3.1), and incorporate bounds on the control.

$$\frac{dx(t)}{dt}=\gamma x(t)+u(t),\;x(0)=x_{0},\;\gamma >0,\;0\leq t\leq 1,\;0\leq u(t)\leq 2.$$

We seek to maximize a cost function *J* that is linear in *u*,

$$J=\int_{0}^{1}(ax^{2}-bu)\;dt,\;a>0,\;b>0.$$

We form the Hamiltonian and derive the co-state equation and transversality condition

$$H=ax^{2}-bu+\lambda (\gamma x+u).$$

The co-state equation is

$$\frac{d\lambda }{dt}=-\frac{\partial H}{\partial x}=-2ax-\lambda \gamma ,$$

with transversality condition *λ*(1) = 0.

In seeking the optimality condition, we find

3.6 $$\frac{\partial H}{\partial u}=\lambda -b.$$

As equation (3.6) does not depend on *u*, we define a switching function

ψ(t)=λ−b,

and produce an expression for the control, based on the bounds on *u* and the sign of the switching function:

3.7 $$u^{*}(t)=\left\{ \begin{array}{cc} 0, & \psi (t)<0,\\ 2, & \psi (t)>0. \end{array}\right.$$

**Figure 4.**
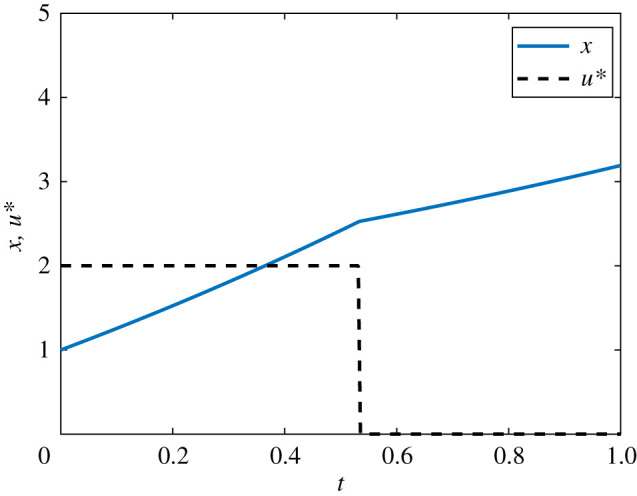
Solution to the linear bang-bang control problem. The optimal control, u*(*t*), is shown in black dash and the corresponding state, *x*(*t*), in blue. This solution is produced with model parameter *γ* = 0.5, time-step d*t* = 3.91 × 10^−3^, over the interval 0 ≤ *t* ≤ 1, with pay-off weightings of *a* = 1 for the state, and *b* = 3 for the control. The bang-bang control has prescribed bounds of 0 ≤ u*(*t*) ≤ 2.

If *ψ*(*t*) is zero over any finite interval excluding isolated points, the optimal control is singular rather than bang-bang. Over such intervals, minimization of the Hamiltonian does not provide sufficient information to determine the optimal control, and further conditions must be considered [38,57]. We restrict our focus in this work to non-singular bang-bang optimal control problems. The numerical solution to the linear bang-bang control problem is presented in figure 4. Convergence to this solution via the FBSM required N=8 iterations.

#### 3.1.3. Continuous control with fixed endpoint

For the fixed endpoint problem, we proceed with the same state equation; however we now impose a terminal condition on *x*.

$$\frac{dx(t)}{dt}=\gamma x(t)+u(t),\;x(0)=x_{0},\;x(1)=10,\;\gamma >0,\;0\leq t\leq 1.$$

**Figure 5.**
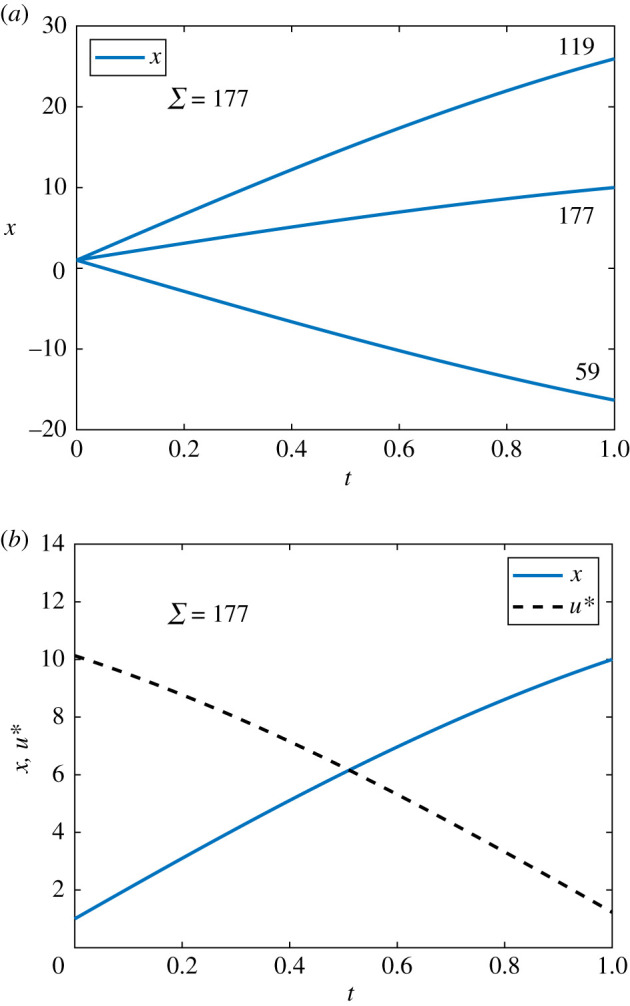
Results are presented for the linear problem with specified terminal state value, *x*(*t*_*N*_) = 10, solved using the adapted FBSM. Underlying FBSM problems are solved with time-step d*t* = 3.91 × 10^−3^, over the interval 0 ≤ *t* ≤ 1, with pay-off weightings of *a* = *b* = 1. In (*a*), the *x*(*t*) iterates of the adapted FBSM are presented. We annotate the cumulative function evaluations after the first ($N^{\left(1\right)}=59$) and second ($N^{\left(1\right)}+N^{\left(2\right)}=119$) iterations of the adapted FBSM, based on initial guesses for *λ*(*t*_*N*_) of *λ*(*t*_*N*_) = −10 and *λ*(*t*_*N*_) = 10. The total cumulative function evaluations required for convergence of the adapted FBSM, $\Sigma =N^{\left(1\right)}+N^{\left(2\right)}+N^{\left(3\right)}=177$, is indicated. The converged result for *x*(*t*), satisfying $|x(t_{N})-10|$ ≤ 1 × 10^−10^ is presented in (*b*); this figure also includes the optimal control, u*(*t*).

We seek to maximize the same quadratic cost function *J*, as considered in equation (3.2). As such, we form the same Hamiltonian given in equation (3.3) and derive the same co-state, equation (3.4), and expression for the control, equation (3.5). Note however that we do not prescribe a final time condition on the co-state equation via the transversality condition; as the system already has two boundary conditions, doing so would cause it to be overdetermined. Instead, we make two guesses for *λ*(1); for example, *λ*^(0)^(1) = −10 and *λ*^(1)^(1) = 10. We proceed by applying the adapted FBSM outlined in §2, using these guesses to initialize the secant method. Numerical results for the linear fixed endpoint control problem are presented in figure 5. Convergence of the adapted FBSM is achieved after Σ=177 iterations.

### 3.2. Multiple-variable nonlinear model

The AML model is a nonlinear coupled multi-species model describing the interactions between progenitor blood cells, *A*(*t*), and leukaemic stem cells, *L*(*t*), that occupy the same niche in the bone marrow, thereby competing for space and resources. Haematopoietic stem cells, *S*(*t*), act as upstream production of *A*(*t*). These dynamics have been explored in the literature both experimentally [58,59], and through mathematical modelling [52,60]. We subject the model to a chemotherapy-like control, *u*(*t*), that acts as an additional death term for *L*(*t*). The state can be expressed as ***x***(*t*) = [*S*(*t*), *A*(*t*), *L*(*t*)]^*T*^. As there are now three state equations, we require three co-state equations: ***λ***(*t*) = [*λ*_1_(*t*), *λ*_2_(*t*), *λ*_3_(*t*)]^*T*^. We suppress the explicit time dependence of the state and co-state variables and the control in the following equations for notational convenience:

3.8 $$\begin{array}{rl} & \frac{dS}{dt}=\underset{\mathrm{logistic}\;\mathrm{growth}}{\underbrace{\rho_{S}S(1-S)}}-\underset{\mathrm{differentiation}}{\underbrace{\delta_{S}S}},\\ & \frac{dA}{dt}=\underset{\begin{array}{c} \mathrm{upstream}\;\\ \mathrm{production} \end{array}}{\underbrace{\delta_{S}S}}+\underset{\begin{array}{c} \mathrm{logistic}\;\mathrm{growth}\;\\ \mathrm{with}\;\mathrm{competition} \end{array}}{\underbrace{\rho_{A}A(1-A-L)}}-\underset{\mathrm{differentiation}}{\underbrace{\delta_{A}A}}\\ \mathrm{and}\; & \frac{dL}{dt}=\underset{\begin{array}{c} \mathrm{logistic}\;\mathrm{growth}\;\\ \mathrm{with}\;\mathrm{competition} \end{array}}{\underbrace{\rho_{L}L(1-A-L)}}-\underset{\mathrm{differentiation}}{\underbrace{\delta_{L}L}}-\underset{\begin{array}{c} \mathrm{immune}\;\\ \mathrm{response} \end{array}}{\underbrace{\frac{\alpha L}{\gamma +L}}}-\underset{\begin{array}{c} \mathrm{chemotherapy}\;\\ \mathrm{control} \end{array}}{\underbrace{uL}}. \end{array}\}$$

For each control problem associated with the AML model, we use initial conditions that yield a coexisting steady state in the absence of control (all three species non-zero): *S*(0) = 1 − *δ*_*S*_/*ρ*_*S*_, *A*(0) = 0.3255 and *L*(0) = 0.3715. We solve the AML problems numerically on the domain 0 ≤ *t* ≤ 10, with time-step d*t* = 4.88 × 10^−4^, giving *N* = 20481 time points. Model parameters are specified in table 1.

**Table 1.** AML model parameters. Parameters correspond to those presented with the original model [52], with immune response parameters introduced in subsequent work [5].

description	variable	value	dimension
proliferation of *S*	*ρ*_*S*_	0.5	[T^−1^]
proliferation of *A*	*ρ*_*A*_	0.43	[T^−1^]
proliferation of *L*	*ρ*_*L*_	0.27	[T^−1^]
differentiation of *S* into *A*	*δ*_*S*_	0.14	[T^−1^]
differentiation of *A*	*δ*_*A*_	0.44	[T^−1^]
differentiation of *L*	*δ*_*L*_	0.05	[T^−1^]
characteristic rate of the immune response	*α*	0.015	[T^−1^]
half-saturation constant of the immune response	*γ*	0.1	[−]

#### 3.2.1. Continuous control

For the AML continuous control problem, we seek to minimize a quadratic cost function *J* that accounts for both the cost of applying the control and the cost of the leukaemic burden, subject to

3.9 $$J=\int_{0}^{10}(a_{1}u^{2}+a_{2}L^{2})dt,\;a_{1}>0,a_{2}>0.$$

We form the Hamiltonian and derive the co-state equation, transversality condition and optimality condition. The Hamiltonian is given by

3.10 $$\begin{array}{rl} H & =a_{1}u^{2}+a_{2}L^{2}+(\rho_{S}S(1-S)-\delta_{S}S)\lambda_{1}\\ & \;+(\delta_{S}S+\rho_{A}A(1-A-L)-\delta_{A}A)\lambda_{2}\\ & \;+(\rho_{L}L(1-A-L)-\delta_{L}L-\frac{\alpha L}{\gamma +L}-uL)\lambda_{3}. \end{array}$$

The co-state equations are

3.11 $$\begin{array}{rl} \frac{d\lambda_{1}}{dt}=-\frac{\partial H}{\partial S}= & -\rho_{S}\lambda_{1}+2\rho_{S}\lambda_{1}S+\delta_{S}\lambda_{1}-\delta_{S}\lambda_{2},\\ \frac{d\lambda_{2}}{dt}=-\frac{\partial H}{\partial A}= & -\rho_{A}\lambda_{2}+2\rho_{A}\lambda_{2}A+\rho_{A}\lambda_{2}L+\delta_{A}\lambda_{2}+\rho_{L}\lambda_{3}L\\ \mathrm{and}\;\;\;\frac{d\lambda_{3}}{dt}=-\frac{\partial H}{\partial L}= & -2a_{2}L+\rho_{A}\lambda_{2}A-\rho_{L}\lambda_{3}+\rho_{L}\lambda_{3}A+2\rho_{L}\lambda_{3}L\\ & +\delta_{L}\lambda_{3}+\frac{\alpha \gamma \lambda_{3}}{{\left(\gamma +L\right)}^{2}}+\lambda_{3}u, \end{array}\}$$

with transversality conditions *λ*_1_(10) = *λ*_2_(10) = *λ*_3_(10) = 0, obtained in the usual way. In this case, the optimality condition is

3.12 $$\frac{\partial H}{\partial u}=2a_{1}u-\lambda_{3}L=0,$$

such that the optimal control is given by

3.13 $$u^{*}(t)=\frac{\lambda_{3}L}{2a_{1}}.$$

**Figure 6.**
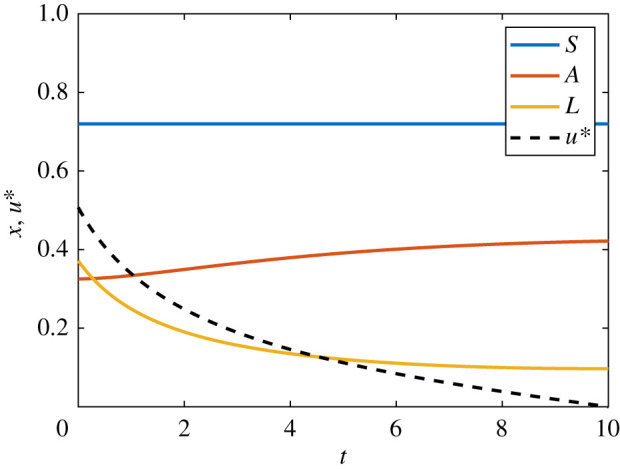
Solution to the AML continuous control problem. The optimal control, u*(*t*), is shown in black dash and the corresponding state equations for *S*(*t*), *A*(*t*) and *L*(*t*) are shown in blue, red and yellow, respectively. This solution is produced with model parameters given in table 1, time-step d*t* = 4.88 × 10^−4^, over the interval 0 ≤ *t* ≤ 10, with pay-off weightings of *a*_1_ = 1 for the control, and *a*_2_ = 2 for state variable *L*(*t*).

Numerical solutions for the AML continuous control problem are presented in figure 6. These solutions are obtained via the FBSM, requiring N=38 iterations with *ω* = 0.55. This choice of *ω* minimizes N for the AML continuous control problem solved with the FBSM without acceleration techniques. We discuss the choice of *ω* further in §5.

#### 3.2.2. Bang-bang control

For the bang-bang AML problem, we consider the same states as in equation (3.8), and incorporate bounds, 0 ≤ *u* ≤ 0.3, on the control. We seek to minimize a cost function *J* that is linear in the control and the state variable *L*:

3.14 $$J=\int_{0}^{10}(a_{1}u+a_{2}L)\;dt,\;a_{1}>0,a_{2}>0.$$

We form the Hamiltonian and derive the co-state equations, transversality conditions and optimality condition. The Hamiltonian is given by

3.15 $$\begin{array}{rl} H & =a_{1}u+a_{2}L+(\rho_{S}S(1-S)-\delta_{S}S)\lambda_{1}\\ & \;+(\delta_{S}S+\rho_{A}A(1-A-L)-\delta_{A}A)\lambda_{2}\\ & \;+(\rho_{L}L(1-A-L)-\delta_{L}L-\frac{\alpha L}{\gamma +L}-uL)\lambda_{3}. \end{array}$$

The co-state equations are

$$\begin{array}{rl} \frac{d\lambda_{1}}{dt}= & -\rho_{S}\lambda_{1}+2\rho_{S}\lambda_{1}S+\delta_{S}\lambda_{1}-\delta_{S}\lambda_{2},\\ \frac{d\lambda_{2}}{dt}= & -\rho_{A}\lambda_{2}+2\rho_{A}\lambda_{2}A+\rho_{A}\lambda_{2}L+\delta_{A}\lambda_{2}+\rho_{L}\lambda_{3}L\\ \mathrm{and}\;\frac{d\lambda_{3}}{dt}= & -a_{2}+\rho_{A}\lambda_{2}A-\rho_{L}\lambda_{3}+\rho_{L}\lambda_{3}A+2\rho_{L}\lambda_{3}L\\ & +\delta_{L}\lambda_{3}+\frac{\alpha \gamma \lambda_{3}}{{\left(\gamma +L\right)}^{2}}+\lambda_{3}u, \end{array}\}$$

with transversality conditions *λ*_1_(10) = *λ*_2_(10) = *λ*_3_(10) = 0. In this case, the switching function is

3.16 $$\psi (t)=\frac{\partial H}{\partial u}=a_{1}-\lambda_{3}L,$$

such that the optimal control is given by

3.17 $$u^{*}(t)=\left\{ \begin{array}{cc} 0, & \psi (t)>0,\\ 0.3, & \psi (t)<0. \end{array}\right.$$

**Figure 7.**
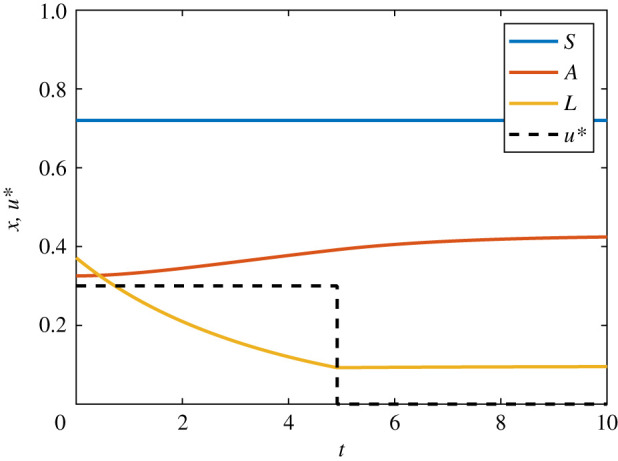
Solution to the AML bang-bang control problem. The optimal control, u*(*t*), is shown in black dash and the corresponding state equations for *S*(*t*), *A*(*t*) and *L*(*t*) are shown in blue, red and yellow, respectively. This solution is produced with model parameters given in table 1, time-step d*t* = 4.88 × 10^−4^, over the interval 0 ≤ *t* ≤ 10, with pay-off weightings of *a*_1_ = 1 for control, and *a*_2_ = 2 for the state variable *L*(*t*).

Note that the correspondence between the sign of *ψ*(*t*) and the chosen bound is reversed in equation (3.17) relative to equation (3.7) as we are now performing minimization rather than maximization. Numerical solutions for the AML bang-bang control problem are presented in figure 7. These solutions are obtained via the FBSM, requiring N=34 iterations with *ω* = 0.4. This choice of *ω* minimizes N for the AML bang-bang control problem solved with the FBSM without acceleration techniques. We discuss the choice of *ω* further in §5.

#### 3.2.3. Continuous control with fixed endpoint

For the fixed endpoint problem, we proceed with the same state equations as for the AML continuous control problem given in equation (3.8); however we now impose a terminal condition on the leukaemic population: *L*(10) = 0.05. We seek to minimize the same quadratic cost function *J*, as considered in equation (3.9). We form the same Hamiltonian given in equation (3.10) and derive the same co-state, equation (3.11), and expression for the control, equation (3.13).

**Figure 8.**
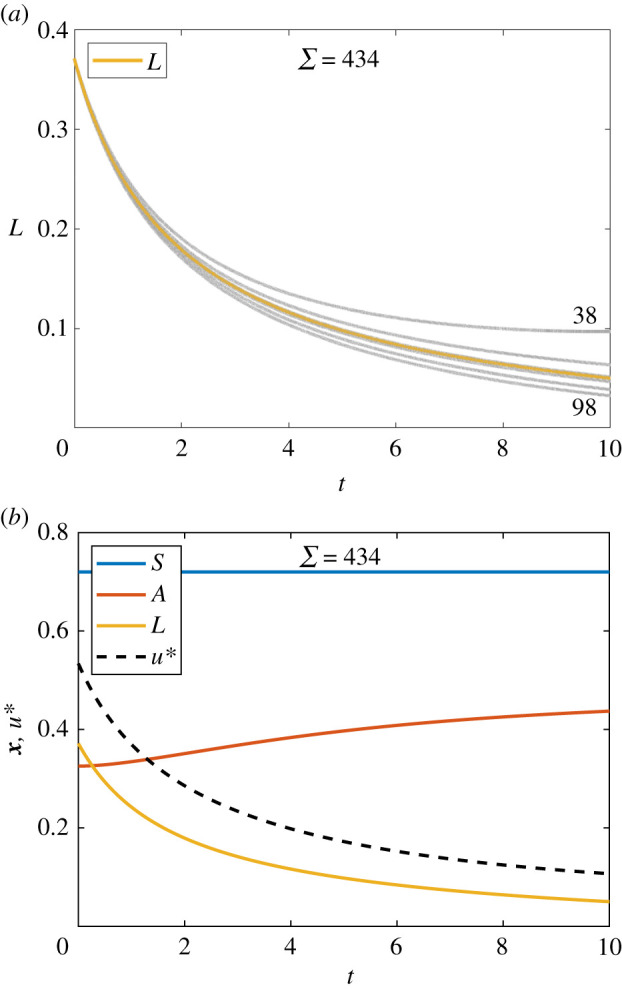
Results are presented for the AML problem with specified terminal state, *L*(*t*_*N*_) = 0.05, solved using the adapted FBSM. Each underlying FBSM problem is solved with model parameters given in table 1, time-step d*t* = 4.88 × 10^−4^, over the interval 0 ≤ *t* ≤ 10, with pay-off weightings of *a*_1_ = 1 for the control, and *a*_2_ = 2 for the state variable *L*(*t*). In (*a*), the *L*(*t*) iterates of the adapted FBSM are presented in grey; the converged solution satisfying *L*(*t*_*N*_) = 0.05 is plotted in yellow. We annotate N for the first ($N^{\left(1\right)}=38$) and second ($N^{\left(1\right)}+N^{\left(2\right)}=98$) iterations of the adapted FBSM, based on initial guesses for *λ*_3_(*t*_*N*_) of *λ*_3_(*t*_*N*_) = 0 and *λ*_3_(*t*_*N*_) = 10. Due to the close proximity, subsequent iterations are not annotated. The cumulative function evaluations required for convergence of the adapted FBSM (Σ=434) is indicated. The converged result for *L*(*t*), satisfying |*L*(*t*_*N*_) − 0.05| ≤ 1 × 10^−10^, is presented in (*b*); this figure also includes the optimal control, u*(*t*), and trajectories for *S*(*t*) and *A*(*t*).

We obtain final time conditions, *λ*_1_(10) = *λ*_2_(10) = 0, via the transversality conditions as usual; however we do not prescribe *λ*_3_(10). Instead, we make two guesses for *λ*_3_(10); for instance, $\lambda_{3}^{\left(0\right)}(10)=0$ and $\lambda_{3}^{\left(1\right)}(10)=10$. We then proceed by applying the adapted FBSM outlined in §2, using these guesses to initialize the secant method. Numerical results for the AML fixed endpoint control problem are presented in figure 8. These results are produced using the adapted FBSM with *ω* = 0.55 in Σ=434 iterations. This choice of *ω* minimizes Σ for the AML fixed endpoint control problem solved with the FBSM without acceleration techniques. We discuss this further in §5.

## 4. Iterative accelerators

In this section, we outline several techniques for acceleration of iterative schemes. Where appropriate, we first present the univariate/scalar version of the method for familiarity, then provide the multivariate/vector analogue of the method for use with accelerating the FBSM. We attempt to use notation that aligns most closely with commonly used notation in the literature, while maintaining internal consistency in this work. In the scalar case, we consider the iterative process *x*^(*k*+1)^ = *f*(*x*^(*k*)^), where *x*^(*k*)^ is the *k*th iterate and *f* is the iterating function. In the vector case, we consider *X*^(*k*+1)^ = *F*(*X*^(*k*)^), where $X^{\left(k\right)}={\left[x_{0}^{\left(k\right)},x_{1}^{\left(k\right)},\ldots ,\;x_{N}^{\left(k\right)}\right]}^{T}$ is the *k*th iterate, consisting of *N* + 1 values, and *F* = [*f*_0_, *f*_1_, …, *f*_*N*_]^T^ is the *N* + 1 dimensional operator of the iterative process. For clarity, we stress that in the context of the acceleration algorithms applied to the FBSM, *X*^(*k*)^ is the discretized control in the *k*th iteration.

The acceleration methods considered in this work apply either to problems stated as fixed point iterations (as above), or as root-finding problems. For acceleration via root-finding algorithms, we can consider the complementary problems in the scalar and vector setting, respectively: *g*(*x*) : = *x* − *f*(*x*) = 0 and *G*(*X*) : = *X* − *F*(*X*) = **0**, where **0** is the zero column vector of length *N* + 1.

We note that many of the methods presented here can be written in several different forms. While some forms better facilitate analysis of aspects such as convergence speed and numerical stability, others emphasize ease of understanding and implementation. In this work, we prioritize usability and present methods and algorithms in forms reflective of their implementation where possible. For the purpose of this work, we feel it is sufficient to present the methods and discuss their implementation without delving into their derivation or rigorous theoretical convergence results. For readers interested in these aspects, we suggest these articles [61,62], and numerical analysis texts [63,64].

### 4.1. Newton and quasi-Newton methods

Newton’s method is one of the most prevalent root-finding algorithms, due to its relatively straightforward implementation and potential for quadratic convergence [64]. For a univariate function, Newton’s method is given by

4.1 $$x^{\left(k+1\right)}=x^{\left(k\right)}-\frac{\;f\;(x^{\left(k\right)})}{f^{\prime }(x^{\left(k\right)})}.$$

We arrive at the scalar secant method by replacing the derivative term, *f*′(*x*^(*k*)^), in equation (4.1) with a finite difference approximation

$$x^{\left(k+1\right)}=x^{\left(k\right)}-f\;(x^{\left(k\right)})\frac{x^{\left(k\right)}-x^{\left(k-1\right)}}{f\;(x^{\left(k\right)})-f\;(x^{\left(k-1\right)})}.$$

Newton’s method for multivariate systems is

$$\begin{array}{rl} & X^{\left(k+1\right)}=X^{\left(k\right)}+\Delta X^{\left(k\right)},\\ & \;\text{where}\;\Delta X^{\left(k\right)}\text{is obtained by solving }\\ & J_{k}\Delta X^{\left(k\right)}=-F(X^{\left(k\right)}). \end{array}$$

Here, *J*_*k*_ is the Jacobian matrix of *F* evaluated at *X*^(*k*)^ [64]. Setting aside the interpretation of the Jacobian in the context of the FBSM, numerically approximating an *N* × *N* Jacobian matrix using finite differences requires $O(N^{2})$ FBSM iterations at each Newton step. A range of quasi-Newton methods have been developed to minimize the computational expense associated with computing the Jacobian at each Newton step. It is not immediately apparent how the secant method should be extended to multivariate systems, but one such interpretation is the quasi-Newton Broyden’s method. Broyden’s method reduces the number of function evaluations required at each Newton step by forming the full Jacobian only initially, then updating the Jacobian matrix via a rank-one update based on the secant method [63,65]. We later discuss the Wegstein method [66], which is another interpretation of the secant method in multivariate settings.

In the context of accelerating the FBSM, techniques that require forming or approximating a full Jacobian, even once, are not appropriate. We have an *N* + 1 dimensional system, where *N* + 1 is the number of time points in the discretization of the ODEs, so we expect *N* to be large, relative to the number of iterations required for the FBSM to converge without acceleration techniques, via equation (2.6). As such, we restrict our focus to Jacobian-free methods in the remainder of this section; in particular, we discuss and implement the Wegstein and Aitken–Steffensen methods and Anderson acceleration. We provide a broad overview alongside the key equations here, and provide complete algorithms alongside notes for implementation in §4 of the electronic supplementary material.

### 4.2. Wegstein method

Wegstein’s method can be thought of as an element-wise extension of the secant method to multivariate systems [67]. Although Wegstein’s method appears less popular than other methods considered in this work, it has found practical utility, particularly in chemical and process engineering software [68,69]. We include it here due to the striking similarity it bears to the control update with relaxation presented in equation (2.6). It is also one of the more straightforward techniques, both in conception and implementation:

4.2 $$\begin{array}{c} {\hat{x}}^{\left(k+1\right)}=f\;(x^{\left(k\right)}),\\ x^{\left(k+1\right)}=q^{\left(k\right)}x^{\left(k\right)}+(1-q^{\left(k\right)}){\hat{x}}^{\left(k+1\right)}, \end{array}\}$$

4.3 $$\mathrm{where}\;q^{\left(k\right)}=\frac{a^{\left(k\right)}}{a^{\left(k\right)}-1},\mathrm{and}\;a^{\left(k\right)}=\frac{\;f\;(x^{\left(k\right)})-f\;(x^{\left(k-1\right)})}{x^{\left(k\right)}-x^{\left(k-1\right)}}.$$

In implementation, from an initial value *x*_0_, it is necessary to perform two function evaluations, i.e. *x*_1_ = *f*(*x*_0_), and *f*(*x*_1_), before it is possible to compute equation (4.3) for the Wegstein method [66]. In subsequent iterations only one new function evaluation is required.

The extension of Wegstein’s method to multivariate systems follows exactly the process outlined in equations (4.2) and (4.3), as it is extended element-wise. While convergence is guaranteed when using Wegstein’s method for a single nonlinear equation, the uncoupling implied by the element-wise extension can lead to divergence [70].

In equations (4.2) and (4.3), *q*^(*k*)^ denotes *q* in the *k*th iteration; however, we note that it is not necessarily most effective to update *q* every iteration. As such, in this work, we explore various updating regimes. There is also the option of applying bounds on *q*. Bounds of −5 < *q*_*i*_ < 0, ∀i, where *i* denotes the *i*th element of the system, are frequently applied when implementing Wegstein’s method [71,72]. This bounding appears to work reasonably well for the small nonlinear test systems we consider in §5 of the electronic supplementary material, although we were not able to identify a theoretical result supporting this specific choice. For the control problems we consider, this bounding is not effective, so we apply different bounds, discussed further in §5. The univariate Wegstein method can be thought of as a modification of the Aitken method, which at the time the Wegstein method was developed, was only understood for the univariate case [73].

### 4.3. Aitken–Steffensen method

Aitken’s Δ^2^ method, also referred to as Aitken’s delta-squared process or Aitken extrapolation, was originally posed by Aitken in 1927 as a means of extending Bernoulli’s method of approximating the largest root of an algebraic equation. This extension facilitates numerically approximating not only the largest root, but all roots of the equation [74]. Aitken’s method generates a new sequence, $\hat{x}$, in parallel to the fixed point iteration.

4.4 $$\begin{array}{rl} & {\hat{x}}^{\left(k\right)}=x^{\left(k\right)}-\frac{{\left(x^{\left(k+1\right)}-x^{\left(k\right)}\right)}^{2}}{x^{\left(k+2\right)}-2x^{\left(k+1\right)}+x^{\left(k\right)}},\;\mathrm{or}\\ & {\hat{x}}^{\left(k\right)}=x^{\left(k\right)}-\frac{{\left(\Delta x^{\left(k\right)}\right)}^{2}}{\Delta^{2}x^{\left(k\right)}}, \end{array}$$

where Δ is the difference operator; Δ*x*^(*k*)^ = *x*^(*k*+1)^ − *x*^(*k*)^, and the higher order operator is applied recursively; Δ^2^ *x*^(*k*)^ = Δ(Δ*x*^(*k*)^) = Δ*x*^(*k*+1)^ − Δ*x*^(*k*)^ [64]. From an initial value, *x*^(0)^, two function evaluations, iterations of the underlying fixed point process, must be performed to obtain *x*^(1)^ and *x*^(2)^, before equation (4.4) can be computed.

The derivation of Aitken’s method assumes an underlying linearly converging series of iterates. The order of convergence of the resulting Aitken accelerated series is still linear; however, this series converges faster than the original series [63]. We discuss Aitken’s Δ^2^ method and Steffensen iteration together, as Steffensen iteration is a straightforward extension of Aitken’s method, whereby the Aitken value, ${\hat{x}}^{\left(k\right)}$, is used to continue the fixed point iteration, i.e. $x^{\left(k+1\right)}={\hat{x}}^{\left(k\right)}$. Despite the striking similarity, Steffensen’s method was seemingly developed shortly after (1933) and without knowledge of Aitken’s method [75]. Steffensen iteration can achieve quadratic convergence [64,76,77]. Further theoretical convergence results for the Steffensen method are established by Nievergelt [78] and in a series of papers by Noda [79–81].

Aitken and Steffensen iteration can be extended to multivariate systems [64]. In the following statements, we outline the method for an *N* + 1 dimensional system, $X^{\left(k\right)}={\left[x_{0}^{\left(k\right)},x_{1}^{\left(k\right)},\ldots ,x_{N}^{\left(k\right)}\right]}^{T}\in R^{N+1}$, as appropriate for use with the FBSM

4.5 $${\hat{X}}^{\left(k\right)}=X^{\left(k\right)}-\Delta X^{\left(k\right)}{\left(\Delta^{2}X^{\left(k\right)}\right)}^{-1}\Delta X^{\left(k\right)},$$

where Δ*X*^(*k*)^ = *X*^(*k*+1)^ − *X*^(*k*)^, $X^{\left(k\right)}$ is a matrix constructed with columns (*X*^(*k*)^, *X*^(*k*+1)^, …, *X*^(*k*+*N*)^), such that $X^{\left(k\right)}$ is a square matrix of dimension *N* + 1, with $\Delta X^{\left(k\right)}=X^{\left(k+1\right)}-X^{\left(k\right)}$, and $\Delta^{2}X^{\left(k\right)}=\Delta X^{\left(k+1\right)}-\Delta X^{\left(k\right)}$.

In the form given by equation (4.5), there are glaring issues with using the Steffensen method to accelerate convergence of the FBSM. Setting aside the question of whether $\Delta^{2}X^{\left(k\right)}$ is invertible, forming $X^{\left(k\right)}$ would require O(N) iterations of the FBSM to be performed, and since *N* relates to the number of time points in the discretization of the ODEs in the FBSM, we expect *N* to be large, relative to the number of iterations required for the FBSM to converge without acceleration.

We instead consider a modification of the Steffensen method, requiring fewer function evaluations per iteration. Introduce *m* < *N*, and define Δ*X*^(*k*)^ = *X*^(*k*+1)^ − *X*^(*k*)^ as before, $X^{\left(k\right)}$ is now a rectangular matrix constructed with columns (*X*^(*k*)^, *X*^(*k*+1)^, …, *X*^(*k*+*m*+1)^), such that $X^{\left(k\right)}\in R^{N+1\times m+2}$, with $\Delta X^{\left(k\right)}=X^{\left(k+1\right)}-X^{\left(k\right)}$, and $\Delta^{2}X^{\left(k\right)}=\Delta X^{\left(k+1\right)}-\Delta X^{\left(k\right)}$, both of dimension *N* + 1 × *m*. We now interpret the matrix inverse in equation (4.5) as the Moore–Penrose pseudoinverse [82], a generalization of the matrix inverse for singular and rectangular matrices; we discuss this further in §3 of the electronic supplementary material. This partial implementation requires only *m* + 1 function evaluations per iteration. For the remainder of this document when referring to the Steffensen method we are specifically referring to this partial Steffensen implementation. We present the derivation of the multivariate Aitken–Steffensen method and outline where the partial implementation differs in §3 of the electronic supplementary material.

### 4.4. Anderson acceleration

Anderson acceleration or Anderson mixing, originally denoted as the extrapolation algorithm by Anderson in the 1960s [83], is a technique developed for accelerating convergence of fixed point iteration problems with slowly converging Picard iterations [84]. Anderson acceleration is of particular interest in this work, as it has recently been implemented to accelerate the convergence of a regularized version of the FBSM [48]. In contrast to a standard fixed point iteration, whereby the next iterate depends only on the immediately preceding iterate, Anderson acceleration has ‘memory’ through the inclusion of previous iterates [85]. Unlike other methods considered in this work, Anderson acceleration explicitly uses the differences between residuals of subsequent iterates alongside iterates and their differences in computing future iterates.

Anderson acceleration involves solving a least-squares problem at each iteration. The problem can be expressed in both constrained and unconstrained forms, with the updating step dependent on the form [86,87]. We solve the following unconstrained least-squares problem in each iteration of Anderson acceleration:

4.6 $$\gamma =\underset{\gamma }{\arg\min}(∥G-\gamma dG∥),$$

where arg min( · ) returns the argument, *γ*, that minimizes the expression in equation (4.6). The corresponding updating step is

4.7 $$X^{\left(k+1\right)}=X^{\left(k\right)}+G^{\left(k\right)}-(dX^{\left(k-1\right)}+dG^{\left(k-1\right)})\gamma ,$$

where *G*^(*k*)^ = *F*(*X*^(*k*)^) − *X*^(*k*)^ is the residual, d*X*^(*k*)^ is a matrix with columns (Δ*X*^(*k*−*m*)^, Δ*X*^(*k*−*m*+1)^, …, Δ*X*^(*k*)^), and d*G*^(*k*)^ is a matrix with columns (Δ*G*^(*k*−*m*)^, Δ*G*^(*k*−*m*+1)^, …, Δ*G*^(*k*)^), and *m* indicates the number of previous iterates that are incorporated.

### 4.5. Acceleration methods applied to typical fixed point problems

As a precursor to implementing these acceleration methods for control problems, we apply them to solve example nonlinear systems of dimension 2 × 2, 3 × 3 and 4 × 4. We provide these systems and the results of the acceleration methods compared to standard fixed point iteration in §5 of the electronic supplementary material. We do not discuss these results in detail, although broad comparisons regarding the application of the acceleration methods to these systems and to control problems are made in §6. We provide code on GitHub (https://github.com/Jesse-Sharp/Sharp2021) for implementing the acceleration algorithms to solve systems of arbitrary size.

## 5. Acceleration results

In this section, we discuss the results of applying the acceleration algorithms. When discussing results we are solely focused on reducing N, the number of function evaluations required for the control problems to reach convergence; as in all convergent cases we arrive at the same optimal control results. We first discuss the aspects of each method that can be tuned, then outline the results of the standard FBSM with the best choice of *ω* but without acceleration methods applied, to establish a baseline against which to compare the acceleration methods. A detailed suite of results for each control problem and each acceleration method, for various combinations of tuning parameters, is provided in §6 of the electronic supplementary material.

### 5.1. Tuning

Each method we consider has parameters that can be tuned to improve performance for a given problem. For the FBSM without acceleration, we can select *ω* ∈ [0, 1); the parameter that weights the contribution of the control from the previous iteration, and the newly calculated control, to the control used in the next iteration, as stated in equation (2.6). Control problems based on the linear model are able to converge via direct updating, as given in equation (2.5), equivalent to *ω* = 0. Increasing *ω* in this case only serves to increase N, so we do not attempt to tune *ω* when considering the linear model. Using the standard FBSM without acceleration the continuous linear problem requires N=57 while the bang-bang linear problem requires only N=8.

**Figure 9.**
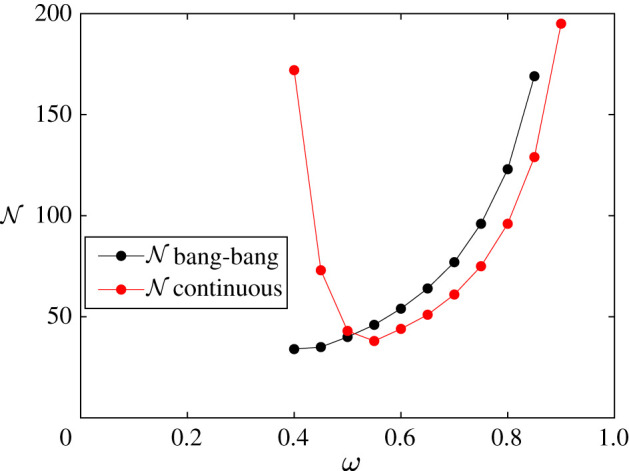
Here, we plot N against *ω* ∈ [0, 1) in increments of 0.05, for the AML continuous (in red) and bang-bang (in black) control problems, using FBSM with no acceleration. Results correspond to model parameters given in table 1, time-step d*t* = 4.88 × 10^−4^, over the interval 0 ≤ *t* ≤ 10. The continuous problem is solved with pay-off weightings of *a*_1_ = 1 for control, and *a*_2_ = 2 for the state variable *L*(*t*), while the bang-bang problem is solved with *a*_1_ = 1 and *a*_2_ = 3. Where an *ω* value does not have a corresponding marker, this indicates that the procedure fails to converge within N=200.

In figure 9, we plot N against *ω* ∈ [0, 1), for the continuous and bang-bang AML problems. As expected, for small *ω* we find that the problem does not converge, and for large *ω*, N increases. For the continuous AML problem we identify *ω* = 0.55 as the best choice, with N=38. For the bang-bang AML problem, we find that *ω* = 0.4 is best, with N=34.

Recall that fixed endpoint problems are solved using the adapted FBSM; this entails solving several control problems to convergence with the FBSM. Each of these control problems can have a different optimal *ω*. In this instance, *ω* = 0.55 also happens to be best for the AML fixed endpoint problem if holding *ω* constant, when considering *ω* ∈ [0, 1) at increments of 0.05. These *ω* values will not coincide in general. When applying the acceleration methods to the fixed endpoint problems, we employ the tuning parameters that perform best for the continuous problem. This does not imply that we are using the best tuning parameters for the acceleration methods in the context of the fixed endpoint problem. Importantly, this demonstrates whether or not the techniques can effectively reduce Σ, the cumulative function evaluations required for convergence of the adapted FBSM for fixed endpoint problems, without requiring prohibitive tuning.

With the Wegstein method, we only select *ω* for the two FBSM iterations required for initialization, and specify *n*, such that we update *q* every *n*th iteration. We generate results for *n* ∈ {1, 2, … , 10}. We also bound *q*; however identifying suitable bounds is challenging. In this work, we select bounds that perform reasonably, but acknowledge that we do not search for optimal bounds, nor do we think that attempting to do so is realistic. This drawback of Wegstein’s method contributes to its inconsistent performance relative to other methods. For the partial Aitken–Steffensen methods, we choose *ω*, and the parameter *m* that specifies the dimension of the *N* + 1 × *m* matrices in the updating step, requiring *m* + 1 function evaluations per iteration. We generate results for *m* ∈ {1, 2, … , 10}. Similarly, for Anderson acceleration we select *ω* and *M*, where *M* determines the maximum number of previous iterations to retain when solving the least-squares problem and performing the updating step. We produce results for *M* ∈ {1, 2, …, 10}.

### 5.2. Wegstein method

For the continuous linear problem, we apply bounds of −2 ≤ *q* ≤ 0. For the bang-bang linear problem, we leave *q* unbounded. For both AML problems, we apply bounds −1 ≤ *q* ≤ 1. We explore the effect of updating *q* every *n*th iteration, *n* ∈ {1, 2, … , 10}. For the continuous linear problem, *n* = 4 minimizes N, although *n* ∈ {1, 2, … , 5} all perform well. For the linear bang-bang problem, the Wegstein method converges without bounding on *q*, and varying *n* does not affect convergence. The Wegstein method outperforms other acceleration methods for the linear bang-bang problem with N=9, but does not improve upon N=8 for the FBSM without acceleration.

For the continuous AML problem, the performance of Wegstein’s method is inconsistent. With *n* = 6 and *ω* = 0.55, the Wegstein method achieves convergence with N=26, outperforming the FBSM; however, almost every other combination of tuning parameters considered with *ω* ∈ [0, 1) and *n* ∈ {1, 2, …, 10} require larger N than the FBSM without acceleration. Generally, increasing *n* produces worse outcomes. We do, however, observe that the Wegstein method can induce convergence for *ω* < 0.4, where the standard FBSM does not converge. For the bang-bang AML problem, the Wegstein method appears more robust; consistently outperforming the standard FBSM across most of the tuning parameter space. The best result requires only N=9, with *ω* = 0 and *n* = 7, although several other combinations of tuning parameters are similarly successful. For *ω* ≥ 0.4, corresponding to values that the underlying FBSM converges, we find that moderate *n* ∈ {3, 4, …, 7} produces the best results; while for smaller *ω*, larger *n* ∈ {6, 7, … , 10} consistently performs best. Once again we observe that convergence is achieved for *ω* values where the underlying FBSM would not converge.

For the linear fixed endpoint problem, the adapted FBSM with the Wegstein method consistently generates a moderate reduction in Σ, compared to the adapted FBSM without acceleration, for all *n* ∈ {1, 2, … , 10}, −2 ≤ *q* ≤ 0. For the AML fixed endpoint problem we do not observe improvement. Using the tuning parameters that perform best for the continuous AML problem, we find that Σ for the adapted FBSM with Wegstein’s method is more than double that of the adapted FBSM without acceleration. This results from the inconsistency of Wegstein’s method with poor tuning. In §6 of the electronic supplementary material, it can be seen that some control problems within the adapted FBSM that require N≈50 without Wegstein’s method, require N≈200 with the specified Wegstein tuning parameters.

### 5.3. Partial Aitken–Steffensen method

Both Aitken and Steffensen methods significantly and consistently outperform the FBSM without acceleration for the continuous linear problem. The Aitken method performs best for *m* ∈ {1, 2, 3}, requiring N=12. Steffensen’s method performs best when *m* = 6, requiring only N=8. In the linear bang-bang case, both Aitken and Steffensen methods perform marginally worse than the FBSM without acceleration, which requires only N=8. In the best cases, with *m* = 1 the Aitken method requires N=10, and with *m* = 7 the Steffensen method requires N=9.

For the continuous AML problem, we observe a stark difference between the Aitken and Steffensen methods; while the Steffensen method is able to achieve convergence for values of *ω* where the underlying FBSM fails to converge, *ω* ≤ 0.35, particularly for *m* ∈ {1, 2, 3, 4}, the Aitken method only converges to the optimal control for *ω* values where the underlying FBSM converges. For *ω* ≤ 0.35, the Aitken method achieves apparent convergence; the iterative procedure terminates as the convergence criteria are met. However, explicitly calculating the pay-off associated with these controls via equation (3.9), and comparing this result to the pay-off associated with the control obtained via the standard FBSM, indicates that the controls obtained via the Aitken method for *ω* ≤ 0.35 are not optimal, as they fail to minimize *J*. The best result for the Aitken method, with *ω* = 0.5 and *m* = 5, requires N=30, marginally improving on the FBSM without acceleration, requiring N=38. Steffensen’s method produces more significant improvements, requiring only N=19 with *ω* = 0.5 and *m* = 5. In each case, neighbouring combinations of tuning parameters also yield equivalent or comparable improvement over the standard FBSM. In the bang-bang AML problem, we observe similar behaviour; for *ω* values that the underlying FBSM fails to converge, the Steffensen method consistently converges. The Aitken method achieves apparent convergence for these values of *ω*; the iterative procedure terminates as the convergence criteria are met; however, the resulting controls contain intermediate values between the lower and upper bounds. As such the resulting controls are not bang-bang, so we treat these results as failing to converge. At best, Aitken’s method requires N=8, with *ω* = 0.5 and *m* = 1, while Steffensen’s requires only N=7, with *ω* = 0.5 and *m* = 5. The vast majority of tuning parameter combinations yield improvements over the N=34 of the standard FBSM.

Aitken and Steffensen methods consistently offer significant improvement over the standard adapted FBSM for the linear fixed endpoint problem for *m* ∈ {1, 2, …, 10}, with the exception of *m* = 1 for the Steffensen method, which yields only marginal improvement. Using the best performing tuning parameters for the continuous AML problem, we find that both Aitken and Steffensen methods improve upon the standard adapted FBSM for the AML fixed endpoint problem. Relative to Σ=434 required without acceleration, the Σ=360 required with Aitken’s method reflects a modest improvement, while the Σ=238 required with the Steffensen method is a significant improvement.

### 5.4. Anderson acceleration

Anderson acceleration performs exceptionally well on the continuous linear problem, requiring only N=7 for *M* ∈ {4, 5, … , 10} compared to N=57 for the standard FBSM. For the linear bang-bang problem, however, it is the worst performing acceleration method; achieving at best N=11, with *M* = 1.

Similarly to the Wegstein and Steffensen methods, Anderson acceleration achieves convergence in both the continuous and bang-bang AML problems for *ω* values where the underlying FBSM fails to converge. Anderson acceleration achieves the best individual result for the continuous AML problem, requiring only N=17, with *ω* = 0.85 and *M* = 6. Again, we observe comparable improvement over a wide range of tuning parameters. For the bang-bang AML problem Anderson acceleration consistently outperforms FBSM without acceleration, particularly for *ω* < 0.7, at best requiring N=17, with *ω* = 0.35 and *M* ∈ {7, 8, 9, 10}, with other non-neighbouring tuning parameter combinations also yielding N=17.

For both the linear and AML fixed endpoint problems Anderson acceleration produces the most significant reduction in Σ, and improves upon the adapted FBSM over a wide range of tuning parameters. In the linear case, Anderson acceleration requires only Σ=24 for *M* ∈ {4, 5, … , 10}. In the AML fixed endpoint problem, using the tuning parameters that perform best for the continuous AML problem, Anderson acceleration converges in only Σ=204; less than half as many as the standard adapted FBSM.

### 5.5. Method comparison with best tuning

**Figure 10.**
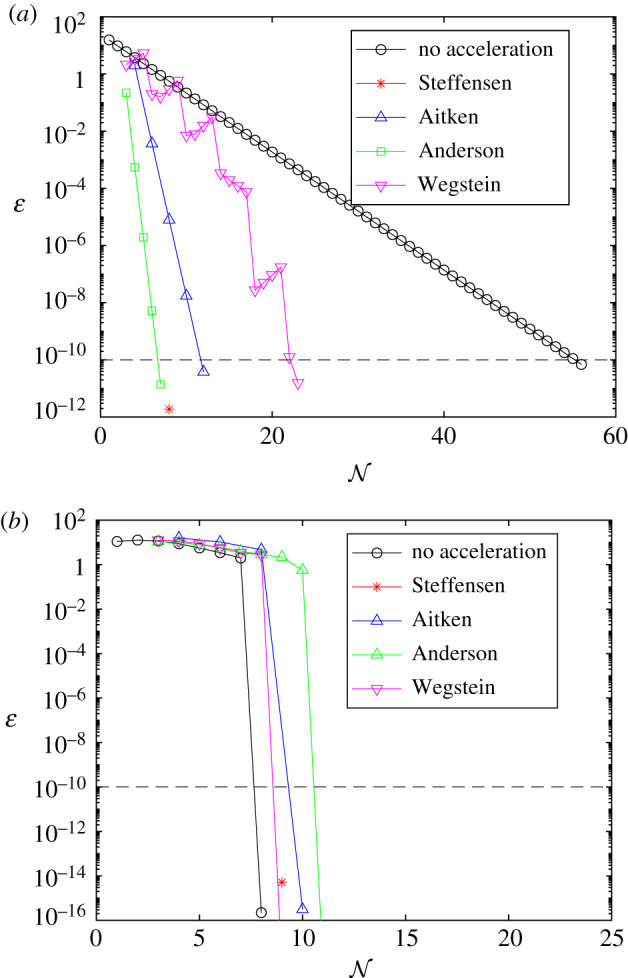
Convergence rates for the result that minimizes N, for each acceleration method, when applied to the linear control problems. Results in this plot are produced with model parameter *γ* = 0.5, time-step d*t* = 3.91 × 10^−3^, over the interval 0 ≤ *t* ≤ 1, with pay-off weighting *a* = *b* = 1 for the continuous control (*a*), and *a* = 1, *b* = 3 for the bang-bang control (*b*). The tolerance of 1 × 10^−10^ required for convergence is marked in black dash. As the methods do not necessarily use the same number of function evaluations per iteration, markers indicate each time *ɛ* is computed. Continuous control results correspond to the FBSM with no acceleration, the partial Steffensen method with *m* = 6, partial Aitken method with *m* = 1, Anderson acceleration with *M* = 4 and Wegstein with bounds −2 ≤ *q* ≤ 0, updating *q* every 4th iteration. Bang-bang results correspond to the FBSM with no acceleration, the partial Steffensen method with *m* = 7, partial Aitken method with *m* = 1, Anderson acceleration with *M* = 1 and Wegstein without bounds on *q*, updating *q* every iteration. The standard FBSM outperformed all acceleration methods in solving the linear bang-bang control problem. We attribute this to how few iterations were required (N=8) for convergence without acceleration.

**Figure 11.**
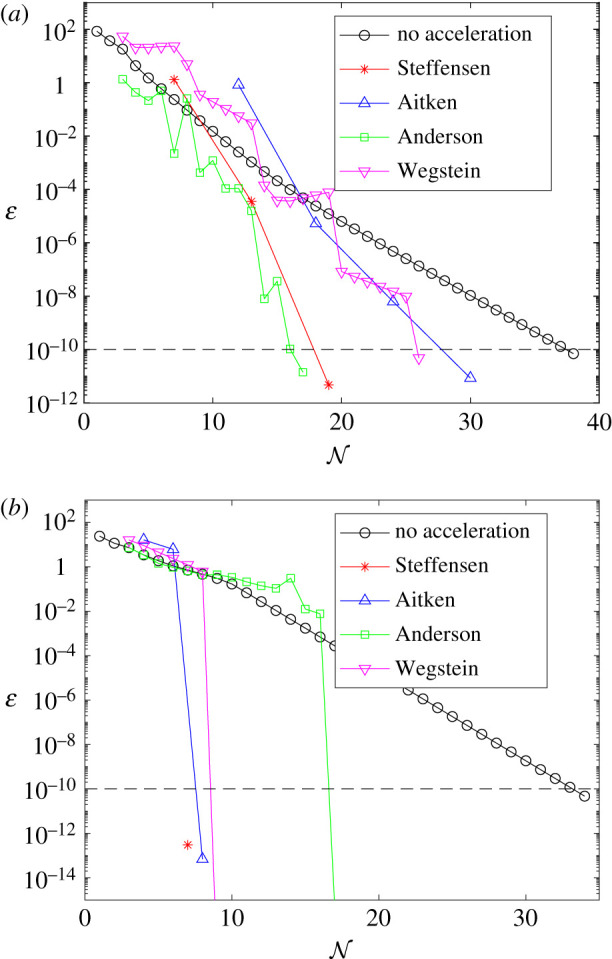
Convergence rates for the converged result that minimizes N, for each acceleration method, when applied to the AML control problems. Results in this plot are produced with model parameter *γ* = 0.5, time-step d*t* = 4.88 × 10^−4^, over the interval 0 ≤ *t* ≤ 1, with pay-off weighting *a* = *b* = 1 for the continuous control (*a*), and *a* = 1, *b* = 3 for the bang-bang control (*b*). The tolerance of 1 × 10^−10^ required for convergence is marked in black dash. As the methods do not necessarily use the same number of function evaluations per iteration, markers indicate each time ɛ is computed. Continuous control results correspond to the FBSM with no acceleration, *ω* = 0.55, the partial Steffensen and partial Aitken methods with *m* = 5 and *ω* = 0.5, Anderson acceleration with *M* = 6 and *ω* = 0.85, and Wegstein method with *ω* = 0.55, bounds −1 < *q* < 1, updating *q* every 6th iteration. Bang-bang results correspond to the FBSM with no acceleration, *ω* = 0.4, the partial Steffensen method with *m* = 5 and *ω* = 0.5, partial Aitken method with *m* = 1 and *ω* = 0.5, Anderson acceleration with *M* = 7 and *ω* = 0.35, and Wegstein method with *ω* = 0, bounds −1 < *q* < 1, updating *q* on the 7th iteration.

Results presented in figures 10 and 11 provide comparison of the error, *ɛ*, as each method approaches convergence, for the linear and AML problems, respectively. Error is measured as the Euclidean norm of the difference between subsequent controls; *ɛ* = ||*F*(*X*^(*k*)^) − *X*^(*k*)^||, with the exception of Aitken’s method, where error is measured as the difference between subsequent values in the Aitken series; $\varepsilon =∥{\hat{X}}^{\left(k\right)}-{\hat{X}}^{\left(k-1\right)}∥$. Convergence is achieved when *ɛ* ≤ 1 × 10^−10^, marked in black dash. In each case, we are plotting the result that minimizes N for each method, over the space of tuning parameters considered, including the best tuning of *ω* for the FBSM without acceleration. Error is plotted on a logarithmic scale. For the linear bang-bang problem with the Wegstein and Anderson methods, and the AML bang-bang problem with the Wegstein method, the error after the final iteration is *ɛ* = 0, as two subsequent iterates for the control are identical. This is represented on the logarithmic scale as a line that intersects the horizontal axis.

## 6. Discussion

Modelling processes in systems biology is complex; frequently involving large state systems consisting of several ODEs [88–90], including canonical examples such as the mitogen-activated protein kinase cascade [91], Wnt/β-catenin signalling pathway [92], early incarnations of whole-cell models [93,94], and other cellular signalling, metabolic and regulatory processes and mechanisms [95,96]. The acceleration methods we implement act only on the control term; the number and form of state equations has no bearing on the mathematical and computational complexity of the acceleration methods. As such, the methods scale excellently with system complexity. In this section, we discuss the results presented in §5, and draw insights into the convergence behaviour of the FBSM when augmented with acceleration techniques. We highlight opportunities for application of these methods, and outline several avenues for further investigation.

### 6.1. Acceleration outcomes

In evaluating the performance of each acceleration method, we are interested in: (1) how significantly they are able to reduce N, (2) method robustness and (3) method accessibility. In this context, we use robustness to refer to how consistently the method outperforms the best tuned FBSM over the range of tuning parameters considered. We judge the accessibility of each method based on implementation and conceptual complexity. Overall, we find that the acceleration methods, particularly Anderson and Steffensen, significantly and robustly reduce N. Anderson acceleration appears most effective for continuous control, while the Steffensen method appears best for bang-bang control. The Aitken method occasionally outperforms Steffensen, but overwhelmingly the Steffensen method appears to be the better option of the two for the range of parameters we consider. Implementing the Anderson and Steffensen methods introduces challenge beyond that of the underlying FBSM, although it is not prohibitively difficult; particularly with reference to the code where we implement these methods, that we make available on GitHub (https://github.com/Jesse-Sharp/Sharp2021). Both methods introduce conceptual complexity, perhaps marginally less so for the Steffensen method due to the similarities it shares with the familiar Newton’s method.

**Figure 12.**
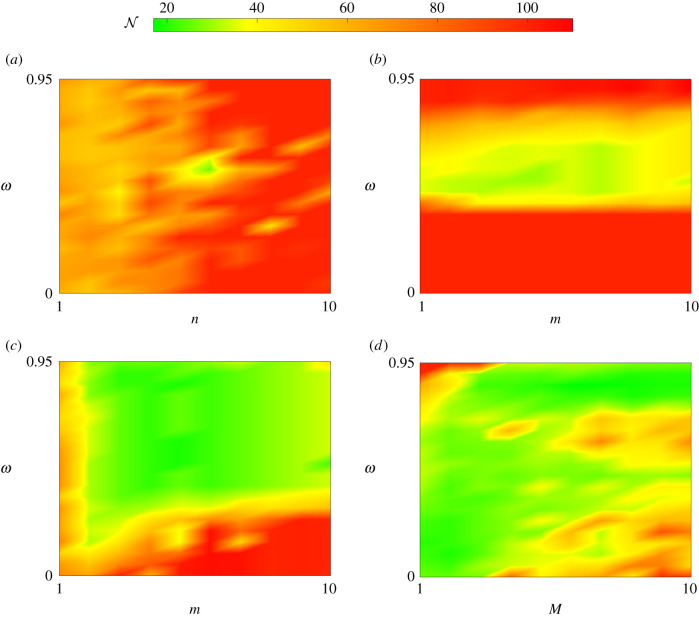
This heatmap provides insight into the convergence behaviour of the acceleration methods for the AML continuous control problem. Here, we visualize N against *ω* and the method specific tuning parameter: *n* for Wegstein (*a*), *m* for partial Aitken (*b*) and partial Steffensen (*c*), and *M* for Anderson acceleration (*d*). Tuning parameter combinations requiring N=38, equivalent to the best tuned FBSM without acceleration, are shaded yellow. Colours in the green-yellow spectrum represent a reduction in N relative to FBSM without acceleration, while colours in the yellow-red spectrum represent an increase in N.

**Figure 13.**
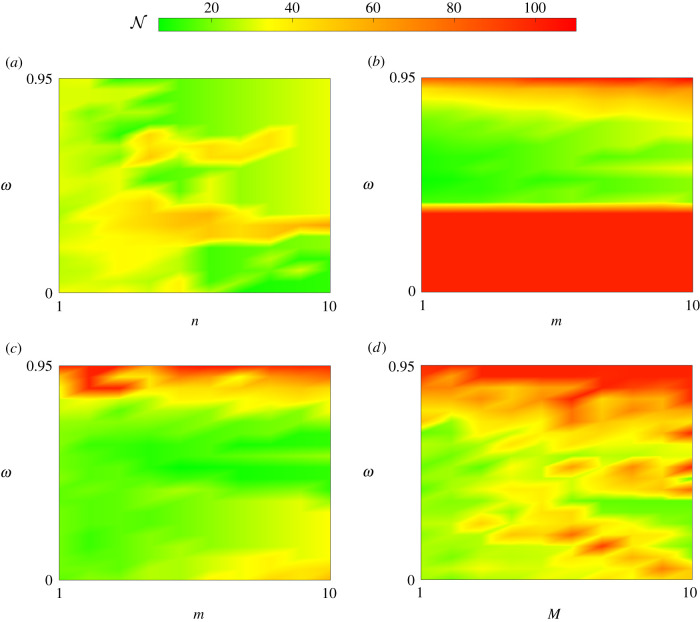
This heatmap provides insight into the convergence behaviour of the acceleration methods for the AML bang-bang control problem. Here, we visualize N against *ω* and the method specific tuning parameter: *n* for Wegstein (*a*), *m* for partial Aitken (*b*) and partial Steffensen (*c*), *M* for Anderson acceleration (*d*). Tuning parameter combinations requiring N=34, equivalent to the best tuned FBSM without acceleration, are shaded yellow. Colours in the green-yellow spectrum represent a reduction in N relative to FBSM without acceleration, while colours in the yellow-red spectrum represent an increase in N.

We produce heatmaps to visualize the convergence behaviour of the acceleration methods across the range of tuning parameters considered. Figure 12 corresponds to the AML continuous control problem, while figure 13 corresponds to the AML bang-bang control problem. Recall that with the tuning of *ω* that minimizes N, the FBSM with no acceleration requires N=38 for the AML continuous control problem, and N=34 for the AML bang-bang control problem. Tuning parameter combinations that reflect a reduction in N relative to the these FBSM results are shaded in the green spectrum, while worse performing combinations are shaded in the red spectrum. The midpoint of the colour spectra, yellow, corresponds to the FBSM result with the best tuning, without acceleration. Simulations are terminated when N exceeds 100; reflecting a combination of tuning parameters that do not yield convergence within this specified maximum. Data supporting these heatmaps, and similar results for the linear control problems are provided in §6 of the electronic supplementary material.

In identifying tuning parameter combinations that yield significant reductions in N, we are looking for bright green areas in the heatmaps. We assess the robustness of each method by considering whether we observe large contiguous areas in the green spectrum, such as in figure 12*c*, indicating robustness, or patchy areas with both green spectrum and red spectrum, such as figure 13*d*, suggesting a lack of robustness.

In table 2, we provide our subjective but informed rating of the methods against the criteria of reduction in N, robustness and accessibility. We consider the continuous and bang-bang control cases separately in terms of reduction in N and robustness.

**Table 2.** Method comparison. We rate the methods considered in this work against key factors such as the reduction in N that they deliver and how robustly they perform over the range of tuning parameters considered, for both continuous (Cts) and bang-bang (BB) control problems. We also consider how accessible the methods are from the standpoints of ease of implementation (Imp) and conceptual complexity. Methods are rated as being either strongly positive (✓✓), positive (✓), neutral (∼), negative (✗) against each aspect.

	reduction in N		robustness		accessibility	
method	Cts	BB	Cts	BB	Imp	complexity
FBSM	∼	∼	✓	✓	✓	✓
Wegstein	∼	✓✓	✗	✓	✓	∼
Aitken	✓	✓	∼	✓	∼	∼
Steffensen	✓✓	✓✓	✓	✓✓	∼	∼
Anderson	✓✓	✓	✓	✓	∼	✗

Despite its conceptual simplicity and straightforward implementation, Wegstein’s method is significantly hampered by the difficulty in choosing bounds. If there were a more informed approach for identifying suitable bounds, Wegstein’s method could be particularly useful for bang-bang control problems. Due to the effect of *ω*, intermediate control iterates of the FBSM do not appear bang-bang; as such the bulk of N are incurred in refining the control about the switching points. Wegstein’s method can accelerate this refinement by adaptively setting *q*_*i*_ = 0 where appropriate.

### 6.2. Convergence insights

As outlined in §5, the linear model control problems converge with *ω* = 0. It may at first seem counterintuitive that Wegstein’s method can improve upon this, given that the computed *q* in Wegstein’s method acts as a stand-in for *ω*. There are two aspects of distinction that enable Wegstein’s method to generate improvement in this case: first, while *ω* is held constant both within the time discretization and between iterations, the element-wise nature of Wegstein’s method enables each element of the discretization to have a different value, *q*_*i*_, *i* ∈ {0, 1, … , *N*}, and *q* can be updated between iterations; second, observing the values of *q*_*i*_ in Wegstein’s method indicates that *q* < 0 can be appropriate. This suggests that *ω* < 0 could also be used to accelerate the standard implementation of the FBSM. Preliminary investigation suggests that this is true for the linear model; however, we do not pursue this further as we expect it to be of limited applicability beyond contrived problems.

**Figure 14.**
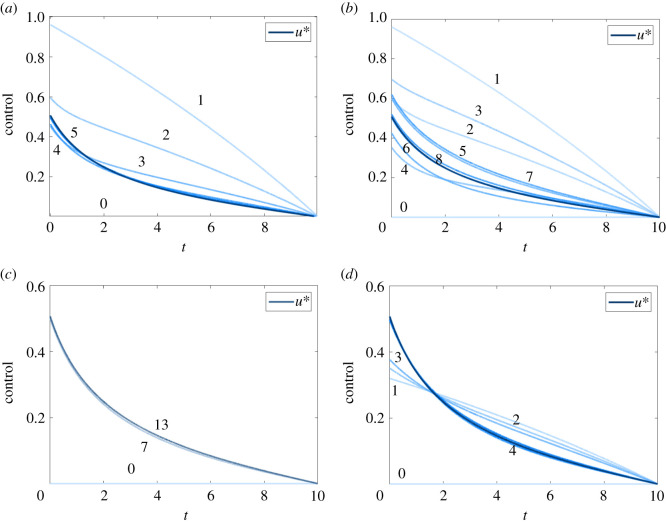
Here we observe the iterates of the control in the AML continuous control problem as it converges, for (*a*) the FBSM with no acceleration, (*b*) the Wegstein method, (*c*) the partial Steffensen method and (*d*) Anderson acceleration. Initial iterates are shown in light blue, while darker blue denotes later iterates. Results for the Aitken method are not shown as they are visually similar to the Steffensen result. We present the results corresponding to the tuning parameters that minimize N, outlined in §5. Where it is visually distinguishable, we indicate the number of function evaluations corresponding to a particular iterate. While all methods produce the same eventual result for u*, they follow considerably different series of iterates. Note that the vertical scale in (*a*) and (*b*) differs from that of (*c*) and (*d*).

We apply the acceleration methods to small nonlinear test systems in §5 of the electronic supplementary material. We know these systems have multiple fixed points; all methods we consider aside from Aitken’s method, in some of our examples, reach different fixed points to fixed point iteration. By contrast, when applied to accelerate control problems, we observe only the Aitken method converging to a result other than the optimal control obtained via the FBSM, as discussed in §5. This apparent convergence of the Aitken method to controls that are not optimal is a significant deterrent to using the Aitken method in situations where the optimal control is not known *a priori*. Outside of this issue with the Aitken method, each acceleration method produces the same optimal control for a given problem. However, they each approach the converged control differently. In figure 14, we plot the control as it converges for the FBSM and acceleration methods. In the code, we provide on GitHub (https://github.com/Jesse-Sharp/Sharp2021), users can view the control iterates of each method as they approach convergence. Visualizing these methods as they converge gives insight into how they may be able to arrive at different fixed points; under certain circumstances the accelerated series of iterates may leave the basin of attraction for the fixed point found via fixed point iteration.

### 6.3. Summary and outlook

In this work, we review the theory and implementation of the FBSM for solving TPBVPs that arise from application of PMP in solving optimal control problems. We study a single-variable linear model and a multiple-variable nonlinear model and consider continuous, bang-bang and fixed endpoint control problems. Conceptualizing the FBSM as a fixed point iteration, we leverage and adapt existing acceleration methods to significantly and robustly increase the convergence rate of the FBSM for a range of optimal control problems. The Anderson and partial Steffensen methods appear to perform best, without requiring prohibitive tuning.

Accelerating the convergence of the FBSM, and reducing the importance of appropriately selecting *ω* for a single control problem, is promising. That said, the real utility of the robust acceleration methods in this work is in application to families of control problems. We provide a glimpse of this benefit through considering fixed endpoint control problems, though other excellent opportunities for application arise due to the uncertainty prevalent in the life sciences. First, it is common for there to be uncertainty around model parameters and structure [97,98]. In this case, solving optimal control problems over several model structures and sets of model parameters provides insight into the sensitivity of the control strategy [99–102]. Secondly, when performing multi-objective optimization, a trade-off is made between objectives. For example, in equation (3.14), we seek to minimize the cumulative negative impact of leukaemia and of the control; parameters *a*_1_ and *a*_2_ weight the relative important of each objective. In practical applications, it is not always clear how to determine these weightings. It can therefore be useful to generate a family of optimal controls that are each optimal for their specific combination of pay-off weighting parameters, akin to a Pareto frontier [49,103,104]. Producing these sets of control results benefits significantly from acceleration techniques such as the Anderson and Steffensen methods, where a consistent reduction in N is obtained without optimal tuning.

In this work, multi-objective optimization is considered in the form of a control problem with a single cost function comprising a scalar combination of state and control terms. More generally, multi-objective optimization can be formulated as a control problem with a vector-valued cost function, with the goal of minimizing each component simultaneously. There are a range of strategies for handling multi-objective optimal control problems formulated in this way, and we direct readers to [105] for a recent and extensive survey.

Here, we have only considered systems subject to a single control. While this is reflective of the vast majority of applications featured in the control literature, there are instances where we are interested in applying multiple controls simultaneously [13,14,51]. The FBSM can be readily applied to solve problems with multiple controls [51]; a logical extension of this work is to adapt the acceleration methods or identify suitable alternative methods for accelerating convergence of the FBSM for problems with multiple controls.

Over a range of tuning parameters the Wegstein, Steffensen and Anderson methods are able to induce convergence where the underlying FBSM fails to converge; such as in the AML control problems with *ω* = 0. This behaviour has been documented for Anderson acceleration [87] and Wegstein’s method [67] when applied to standard fixed point iteration problems. This presents an opportunity for future exploration, in identifying control problems that cannot be solved via the FBSM for any *ω*, and attempting to produce solutions using these acceleration techniques.

The examples we consider in this work include a variety of control problem formulations that arise in systems biology. However, it is worth noting that the examples are not exhaustive. Further challenges can be introduced; either through the formulation of the control problem, or as a result of the behaviour of the underlying system. Examples of such challenges include control problems with singular arcs, path constraints, multiple local solutions, discontinuous dynamics and sensitivity to the initial guess of the control [35]. These challenges can introduce numerical difficulties, and complications in terms of the optimal control theory; for example, control problems with singular arcs typically require additional necessary conditions for optimality beyond those obtained from the PMP [38]. A thorough assessment of the appropriateness of the FBSM as a method for solving control problems with such complications is an avenue for further investigation. We stress that the acceleration techniques that we develop and survey in this work are able to accelerate convergence when compared to a naive FBSM implementation, and in some cases induce convergence where the naive FBSM fails to converge. We anticipate that these trends will persist if these acceleration techniques are applied to appropriately conceived implementations of the FBSM for the various complications outlined here.

## Data accessibility

This article has no additional data.
